# Implementation of a Digital Health Tool for Patients Awaiting Input From a Specialist Weight Management Team: Observational Study

**DOI:** 10.2196/41256

**Published:** 2023-05-31

**Authors:** Petra Hanson, Charlotte Summers, Arjun Panesar, Alexandros Leonidas Liarakos, Dominic Oduro-Donkor, Danniella Whyte Oshodi, Luke Hailston, Harpal Randeva, Vinod Menon, Michaela de la Fosse, Amit Kaura, Emma Shuttlewood, Mark Loveder, Donna Poole, Thomas M Barber

**Affiliations:** 1 Warwick Medical School Coventry United Kingdom; 2 Warwickshire Institute for the Study of Diabetes Endocrinology and Metabolism University Hospitals Coventry and Warwickshire National Health Services Trust Coventry United Kingdom; 3 Digital & Data Driven Research Unit University Hospitals Coventry and Warwickshire National Health Services Trust Coventry United Kingdom; 4 Diabetes Digital Media Health Coventry United Kingdom; 5 National Institute for Health Research Imperial Biomedical Research Centre Imperial College London and Imperial College Healthcare National Health Services Trust London United Kingdom; 6 National Heart & Lung Institute Imperial College London London United Kingdom

**Keywords:** weight management, precision health, digital health, hospital, secondary care, tier 3 weight management, National Health Service, weight, obese, obesity, focus group, perspective, opinion, attitude, behavior change, behavior change, mHealth, mobile health, health app

## Abstract

**Background:**

Digital tools are increasingly used on a population level as a weight loss strategy for people living with overweight and obesity. Evidence supports the feasibility of digital tools for the management of obesity in a community setting, but there is only emerging evidence for the feasibility of such tools in specialist weight management services. No study has assessed the uptake of digital tools among patients awaiting their first appointment with a specialist weight management service.

**Objective:**

The objective of this study was to understand interest, acceptance, and engagement with a digital behavioral change platform to support specialist weight management.

**Methods:**

This was an observational study registered as a service innovation. All patients on the waiting list for a first appointment in the tier 3 weight management service at University Hospitals Coventry and Warwickshire National Health Service (NHS) Trust were eligible to access the NHS-approved digital tool. Data on interest and engagement with the digital tool were collected. Routine clinical data were used to describe patient demographics. Focus groups were held to explore patients’ views on the use of digital tools as part of a specialist weight management service.

**Results:**

A total of 199 patients on the waiting list were informed about the available digital tool. Just over a half (n=102, 51.3%) of patients were interested in using the app, with over one-third (n=68, 34%) of all patients engaging with the app. Overall, a third of patients on the waiting list (n=63, 32%) did not respond to the invite and 34 (17%) of patients expressed no interest in the app. Emotional eating and higher BMI was associated with interest in the Gro Health app. Male gender was associated with reduced engagement with the app. There were no differences in interest in the Gro Health app according to age, ethnicity, metabolic measures of glycemia, and lipid profile.

**Conclusions:**

It is feasible to offer digital tools such as Gro Health to patients awaiting their first appointment with specialist weight management services. Future research should explore barriers and facilitators of engagement with digital tools. Additionally, there is a need to further evaluate the effectiveness of such tools in specialist weight management services.

## Introduction

### Background

Obesity is a leading cause of chronic disease in the 21st century [1]. Despite ongoing research and innovative approaches to prevent and treat obesity, its prevalence continues to increase globally [2]. Our traditional approach to obesity management, including advice on lifestyle changes in real-world settings from health care professionals, is costly and not sustainable, given the increasing demand for health care services. The COVID-19 pandemic highlighted the importance of developing efficient strategies for weight management. With the rates of referrals to our own specialist weight management service at University Hospitals Coventry and Warwickshire (UHCW) National Health Service (NHS) Trust rising by 530% between 2014 (207 patients referred) and 2019 (1319 patients referred), waiting times for referred patients continue to increase. This exacerbates the problem as people most in need are not getting the timely support they so desperately need.

Digital tools have huge potential to transform weight management services. Current evidence shows the emerging effectiveness and weight loss potential of digital health interventions for weight loss in community settings, through the facilitation of positive behavioral changes [3,4]. The application of digital health interventions can result in up to 13% weight loss at 4 months [5] and 7.6% weight loss at 12 months [3]. Indeed, at least in the short term (less than 6 months), such interventions result in greater weight loss than more traditional face-to-face interventions [6], with apparent equivalence in the overall effectiveness between these 2 approaches in the long term (12 months) [3]. Similar findings were observed in a feasibility study of the Low Carb Program app in our obesity service at UHCW, whereby digital tool interventions for diet combined with medical appointments resulted in a similar weight loss to that from a traditionally delivered obesity service [7]. Interestingly, there are no studies that explore the feasibility of offering digital tools to patients on a waiting list for a specialist weight management service, defined as a service comprising specialist dietitians, physicians, and psychologists. This approach may provide initial support and information provision prior to engaging with the hospital obesity service, with the potential to also result in effective weight management in newly referred obese patients. A need for more evidence on this topic was highlighted by a recent meta-analysis by Berry et al [8], who highlighted the need for future studies exploring the effectiveness of digital interventions as an adjunct to specialist weight management services.

Poor uptake and engagement with digital tools remain common challenges with digital health interventions [3,6]. A progressive reduction of user engagement over time may explain a greater weight loss during the initial 6 months of use, with subsequent plateauing of body weight. Within the current literature, there are relatively few studies on how to improve and optimize user take-up and engagement with digital tools, particularly within weight management [9].

Another factor that may contribute to the poor uptake and long-term engagement with digital tools is the specificity of such apps, which generally only address 1 aspect of lifestyle—for instance, the Low Carb Program, which focuses on diet, or Strava, which focuses on exercise. In addition, support for patients from ethnic minorities is usually limited due to apps being available solely in English. Previous evidence has shown that obesity management requires a holistic, health-centered approach [10]. Lifestyle medicine has determined the 6 pillars of lifestyle to be healthy eating, physical activity, restful sleep, stress management, avoidance of risky substances such as alcohol and smoking, and healthy relationships [11]. Additionally, culturally appropriate education has showed consistent benefits over conventional care in terms of metabolic control and condition knowledge [12].

The aim of our study was to investigate the patient demographic and clinical characteristics predictive of expression of interest and subsequent engagement with a digital health weight loss tool among patients referred to tier 3 Specialist obesity service. This study was undertaken as part of a Topol Digital Fellowship funded by Health Education England.

### Objective

Our primary objective was to gauge the general interest in a holistic digital health tool, Gro Health, among newly referred patients awaiting input from our obesity team and to explore the predictors of patient engagement with such digital tools. Our secondary objectives were to gain insight into how to improve the engagement of future patients referred to such digital tools within our obesity service through participant dropout rates and analysis of patient feedback on acceptability and desired features of the digital tool.

## Methods

### Recruitment

We offered access to the NHS-approved digital health tool Gro Health to all patients awaiting their first appointment with our hospital-based (tier 3) specialist weight management team at UHCW between January 2021 and April 2021. All eligible patients were contacted during this period by letter, phone, and email and provided with relevant details about Gro Health app. Those patients who expressed an interest in using the tool were sent an access code to redeem free access and details of how to use the app either via email or post. Patients who were not interested in using the app or did not respond to their initial invite continued to receive usual medical care. All patients who were interested in using the Gro Health app also received the usual clinical care within our obesity service. Therefore, usual clinical care within the obesity service was not influenced by the patient’s interest in the Gro Health app.

### Research Design

This was an observational study, registered as a service evaluation with UHCW research and development department. With clinical data extraction from routine clinical care, formal research ethics committee approval was deemed unnecessary, and no specific consent for this was necessary from patients. Patients did not receive any payment for engagement with the digital tool. Free access to the digital tool was offered to all people on the waiting list as part of the standard of care. Prior to the first use of the digital tool, each person provided informed consent to use the Gro Health app and consent for their anonymized self-reported data to be used for research purposes. No identifiable data were provided from the use of Gro Health app. Patients were invited to participate in a patient engagement workshop (lasting 1 hour) to explore their views on the use of digital tools (both generally and Gro Health app specifically) in specialist obesity services. These were held using Microsoft Teams, and participants were offered an Amazon voucher (£20; US $25) in return for their participation. For the patient engagement workshops, participants provided verbal consent to participate, record the discussion and were reminded of confidential matter of discussion at patient engagement workshops.

### Intervention

Gro Health (Diabetes Digital Media) is an accessible behavior change platform that supports users to self-manage their condition and achieve their self-selected health goals through a holistic approach to health. This encompasses 4 therapeutic areas including mental well-being, sleep, activity, and nutrition. The Gro Health platform facilitates precision digital health by providing evidence-based structured education, guided behavioral change activities, weekly virtual meetups and community support, health tracking, and data-driven insights to users based on their individualized data collected on signup. The user experience is tailored to self-selected health goals, ethnicity, gender, dietary preference, and levels of activity. Gro Health uses the capability, opportunity, motivation, behavior (COM-B) model of behavior change, which identifies 3 factors that need to be present for any behavior to occur: capability, opportunity, and motivation. These factors interact over time so that behavior is seen as part of a dynamic system with positive and negative feedback loops. To create a sustainable behavioral change environment and support users with diverse needs and levels of accessibility, Gro Health is offered across a variety of platforms that include web-based (responsive), iOS, Android, Apple/Google Watch, Smart TV, and digital assistants such as Google Hub and Amazon Alexa in multiple languages (English, French, German, and Hindi) to support the local population. A clinical dashboard enables the clinical team to remotely assess user engagement with the app. A recently reported study demonstrated that the users of Gro Health had improvements in symptoms of stress, anxiety, and depression measured through standardized questionnaires over 12 weeks [13]. Please see Multimedia Appendix 1 for more details on the Gro Health platform’s precision health components.

### Data Collection and Statistical Analysis

Baseline patient and clinical characteristics including age, gender, weight, BMI, ethnicity, blood test results, and psychological surveys were collected as part of routine clinical care in our obesity service. Psychological data collected from patients within our obesity service in the past were used as a control for comparing psychological variables. Patients were categorized into four mutually exclusive groups based on their responses to have free access to the Gro Health app: (1) those who were interested in using the app but did not engage with it, (2) those who were interested in and engaged with the app, (3) those who were not interested in and did not engage with the app, and (4) those who did not respond to the offer of access to the app. App engagement was defined as having opened the app and imputed data within the last month (data collected in August 2021, 4 months after the last person registered with the app). Patients who were not interested in using the digital tool were able to provide a reason for this decision. Patients who registered with the app were asked to complete an anonymized feedback form, using open-ended questions, which was sent to patients via email or were invited to provide feedback and share opinion on using digital tools during patient engagement workshops held between April and May 2021. The workshops were recorded, and the main themes were summarized by the researcher who led these workshops. The hospital lead for patient and public involvement had an oversight of these workshops that complied with the UK standard for public involvement [14]. Formal qualitative analysis from patient engagement workshops was not done as this was outside of the scope of this service evaluation. Themes from workshops contributed to the development of a bespoke digital product. Anonymous data on engagement with the app were analyzed in August 2021. Psychological data collected routinely in our service were used as a control group for comparison of psychological data of newly referred patients who were interested in using the app. Psychological surveys routinely collected consisted of these validated tools: a brief measure for generalized anxiety disorder (GAD-7) [15], a brief depression severity measure (Patient Health Questionnaire-9; PHQ-9) [16], the Warwick-Edinburgh Mental Wellbeing Scale [17], and the Dutch Eating Behavior Questionnaire [18]. A score of ≥10 is considered clinically significant on the GAD-7 and PHQ-9 questionnaires, indicating a likelihood of anxiety and depression.

SPSS (version 27; IBM Corp) and R (R Development Core Team) were used to analyze data. Normal distribution was assessed with Shapiro-Wilk test. Nonparametric data were analyzed with Mann-Whitney *U* test. Parametric data were analyzed with an independent 2-tailed *t* test. A multivariable multinomial logistic regression model was used to evaluate the association between patient characteristics and user groups (interested and engaged, interested and not engaged, refused, and not responded). The reference group for analyses was the interested and engaged group.

## Results

### Descriptive Statistics

**Figure 1:**
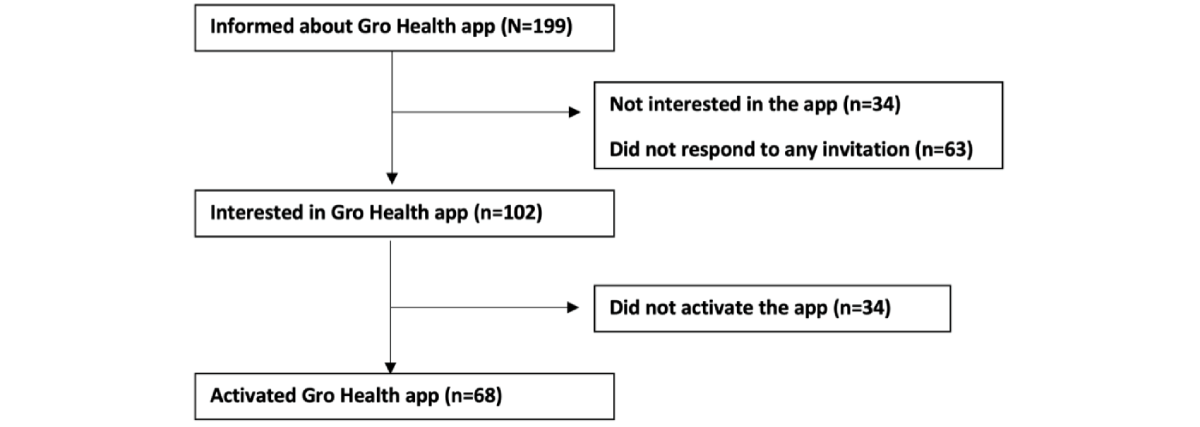
Flowchart of patient inclusion.

All patients awaiting their first appointment with the UHCW obesity team (N=199) were contacted between January and April 2021 and offered free access to the Gro Health app. Figure 1 summarizes the flowchart of study participants. Engagement with the app was assessed in August 2021.

The baseline characteristics of the cohort of patients offered the Gro Health app are summarized in Table 1. All data, except for low-density lipoprotein cholesterol, were not normally distributed.

Just over half (n=102, 51.3%) of patients were interested in using the app, with over one-third (n=68, 34.2%) of these patients engaged with the app. Of the patients who were not interested in using the app, the responses received for their rationale for this decision were categorized as shown in Table 2.

**Table 1:** Baseline characteristics of the whole cohort (N=199).

Characteristics	Values
Age range (years)	18-81
Age (years), median (IQR)	40 (32-51)
BMI (kg/m^2^), median (IQR)^a^	45.5 (41.9-51)
Weight (kg), median (IQR)^b^	130 (114.3-148)
Female, n (%)	154 (77.4)

^a^Data on BMI were available for 193 patients.

^b^Data on body weight were available for 167 patients.

**Table 2:** Reasons for declining an offer to use the Gro Health app among respondents (n=34).

Reasons	Respondents, n (%)
Actively involved in a research trial	7 (21)
Already seen by a weight management clinician	11 (32)
Only surgery wanted or lost weight already	3 (9)
No smartphone or internet	4 (12)
Using other apps	1 (3)
Not interested in apps	4 (12)
Other reasons (died, not happy to tell us the details)	4 (12)

Overall, a third of patients on the waiting list (n=63, 32%) did not respond to communication attempts via telephone, postal letter, or email correspondence. Table 3 summarizes the main characteristics of patients in the 4 groups.

Among patients who were interested in using the app, those not engaged were more likely to be male than those who were engaged (odds ratio 6.17, 95% CI 1.22-31.20; *P*=.03). There were no differences between the user groups according to age or ethnicity. Patients who did not respond were more likely to have a lower BMI when compared to the BMI of patients who were interested and engaged with the app (0.89 kg/m^2^, 95% CI 0.81-1.00; *P*=.05). The results are summarized in Table 4.

**Table 3:** Summary of 4 user groups.

Characteristics		Interested and engaged (n=68)	Interested but not engaged (n=34)	Refused (n=34)	Did not respond (n=63)
Age range (years)		18-71	19-81	21-76	19-69
Age (years), median (IQR)		39 (31-48)	47 (36.3-55)	45.5 (33-53)	38 (31-52)
BMI (kg/m^2^), median (IQR)		46 (41.9-50.5)	45.3 (41.9-49.8)	45.6 (41.6-52.8)	45 (42-50.6)
Weight (kg), median (IQR)		128.3 (112.8-143)	130 (116-149.2)	130 (111.4-144.9)	134 (120-151.4)
**Sex**					
	Female, n (%)	59 (87)	23 (77)	26 (76)	46 (73)
	Male, n	9	11	8	17
**Ethnicity, n (%)**					
	Any Black background	1 (1)	2 (6)	0 (0)	0 (0)
	White	46 (68)	19 (56)	26 (76)	40 (63)
	Any Asian background	2 (3)	2 (6)	1 (3)	3 (5)
	Other/no response	19 (28)	11 (32)	7 (21)	20 (32)

**Table 4:** Association between patient demographics and user groups.

Characteristics		Interested and engaged (n=68)			Interested but not engaged (n=34)			Refused (n=34)			Did not respond (n=63)	
		OR^a^	*P* value^b^	OR (95% CI)		*P* value	OR (95% CI)		*P* value	OR (95% CI)		*P* value
Age (years)		Ref	—^c^	1.02 (0.98-1.07)		.26	1.01 (0.97-1.05)		.55	1.02 (0.98-1.05)		.38
Male		Ref	—	6.17 (1.22-31.2)		.03	4.70 (0.85-25.9)		.08	0.94 (0.23-3.74)		.93
**Ethnicity**												
	White	Ref	—	Ref		—	Ref		—	Ref		—
	Other	Ref	—	2.55 (0.66-9.90)		.18	0.97 (0.22-4.23)		.97	1.90 (0.60-6.00)		.28
	No response	Ref	—	1.99 (0.62-6.32)		.25	0.16 (0.02-1.36)		.09	1.38 (0.54-3.50)		.50
Weight (kg)		Ref	—	0.98 (0.93-1.03)		.45	0.96 (0.92-1.01)		.14	1.03 (1.01-1.07)		.06
BMI (kg/m^2^)		Ref	—	1.02 (0.89-1.17)		.78	1.12 (0.97-1.30)		.13	0.89 (0.81-1.00)		.05

^a^OR: odds ratio.

^b^All *P* values were based on multivariable adjusted multinomial regression models, with the reference group being the interested and engaged group.

^c^Not applicable.

### Psychological Data

Overall, 4 standard screening psychological surveys were completed by 41 patients who were interested in using the digital tool. As a control, we used data collected from patients within our obesity service (n=633) who had completed these screening surveys previously.

Three-quarters of patients in the app group scored ≥10 on the PHQ-9 measure (28/37, 76%) compared to the control group (350/633, 55.5%). Just over half of patients (21/38, 55%) and 46.6% (294/633) scored ≥10 on the GAD-7 questionnaire in the app group and control group respectively. Eight of 41 (19%) patients in the app group and 166 of 633 (26.9%) in the control group endorsed thoughts about suicide or self-harm on the PHQ-9.

There were no statistically significant differences in scores between the app and control groups of patients (n=633) for Warwick-Edinburgh Mental Wellbeing Scale, PHQ-9, GAD-7, Dutch Eating Behavior Questionnaire–restrained eating, and Dutch Eating Behavior Questionnaire–external eating. However, compared with the controls, patients interested in using the app had significantly higher scores for the Dutch Eating Behavior Questionnaire–emotional eating (*P*=.01; Table 5).

**Table 5:** Scores for psychological screening surveys.

	WEMWBS^a^, median score (IQR)	GAD-7^b^, median score (IQR)	PHQ-9^c^, median score (IQR)	DEBQ-R^d^, median score (IQR)	DEBQ-e^e^, median score (IQR)	DEBQ-ext^f^, median score (IQR)
Interested in the app (n=41)	39.5 (31.5-44.3)	10.5 (6-16.3)	14 (9.3-18)	29 (24-34.5)	44 (36-51)	31 (25.3-35.5)
Control group (n=633)	40 (33-48)	9 (5-15)	11 (6-17)	29 (23-34)	38 (26-50)	29 (24-35)

^a^WEMWBS: Warwick-Edinburgh Mental Wellbeing Scale.

^b^GAD-7: generalized anxiety disorder-7.

^c^PHQ-9: Patient Health Questionnaire-9.

^d^DEBQ-R: Dutch Eating Behavior Questionnaire–restrained eating.

^e^DEBQ-e: Dutch Eating Behavior Questionnaire–emotional eating.

^f^DEBQ-ext: Dutch Eating Behavior Questionnaire–external eating.

### Metabolic Parameters

Sixty-two of 102 patients (60.8%) who were interested in the app and 29 of the 34 patients (85%) who refused the app had a screening blood test done by August 2021. The mean values of glycated hemoglobin, triglycerides, and low-density lipoprotein cholesterol are summarized in Table 6. There were no statistically significant differences between the metabolic parameters of those patients who were interested in and those who refused the offer of the app.

**Table 6:** Baseline blood test.

	HbA_1c_^a^ (mmol/mol), median (IQR)	TG^b^ (mmol/l), median (IQR)	LDL^c^-cholesterol (mmol/l), mean (SD)
Interested	39 (36-45)	1.8 (1.3-2.2)	2.6 (0.7)
Refused	37 (35-42)	1.8 (1.4-2.3)	2.9 (1)

^a^HbA_1c_: glycated hemoglobin.

^b^TG: triglycerides.

^c^LDL: low-density lipoprotein.

### Engagement With the Gro Health App

Of the 68 patients who registered with the Gro Health app, 62 (91%) remained engaged at follow-up (defined as having opened the app or imputed data within the last month; data assessed in August 2021). Overall, engagement with the app was 60.8% among those who expressed an initial interest (62/102) and 31.2% (62/199) of patients who were offered the app. Overall, the mean duration of engagement with the app was 184.5 (SD 24.55) days. All patients selected a health goal, with a majority (67/68, 98%) selecting weight loss. All patients who engaged with the app also selected a health focus, which flagged the area of the app the user was currently engaged with, from the 4 therapy areas provided in the app. These included mental well-being (32/68, 47%) and nutrition (36/68, 53%).

### Patients’ Input

To understand the thought processes of our newly referred patients regarding the use of digital apps as part of their clinical care, patient and public engagement workshops were held in January, April, and May 2021. Three main topics were discussed: (1) exposure to digital apps as part of weight management, (2) helpful features of existing digital apps, and (3) any desired features that would be helpful to future patients. The notes from the workshop are summarized in Textbox 1. The results from patient engagement workshops were used to create a bespoke version of Gro Health in order to provide a more tailored digital tool for this group of patients.

Additionally, patients who were interested in using the Gro Health app were asked to complete a feedback form regarding their experience. Anonymized feedback was received from 11 participants. The reported reasons for discontinuation of the app included difficulty in quantifying the weight of food, problems integrating the app with other accessories, and forgetting to use the app. The most common goal that participants set on the app was weight loss. The features of the app that were most enjoyed were weekly educational lessons, downloadable behavior change activities and resources, and health tracking. Patients felt that food lessons improved what and how they eat. Most patients who responded (8/11, 73%) thought that the app was of high or very high credibility.

**Summary of patient and public engagement workshop.**

**Exposure to apps**

- The apps that were free were more desirable.
- The fact that the apps are not homegrown and not recommended by their physician and general practitioner makes choosing an app difficult. Recommendations of digital apps from health care professionals would help to improve confidence.
- Other departments such as physiotherapy have incorporated web-based materials (website and an app) in their services that boost patients’ confidence in their use.

**Helpful features of existing apps**

- Sets goals for them including carb counting, fat, and proteins
- Food tracking and respective nutritional information
- Glucose monitoring
- Explanation of food groups
- Conflates data from other apps

**What the patient would want in an app**

- They would want 1 app or product tailored to all needs
- Recommended menus
- Sends orders to supermarkets
- Simple
- Improved clarity in the instructions to patients
- Recommended or prescribed by their physician or general practitioner

## Discussion

### Principal Findings

We report on the first assessment of interest and engagement of patients awaiting input from a hospital-based obesity service with the digital tool Gro Health. There was significant interest from patients who were referred into NHS weight management services to use a digital tool to support their weight management journey. Emotional eating and higher BMI were associated with interest and engagement with the Gro Health app. This could be explained by an increased desire for additional support tools among those with higher likelihood of emotional eating and higher BMI. Men were less likely to engage with the Gro Health app than women. However, we did not identify any other predictors of patient interest in the digital app, such as ethnicity, age, or metabolic measures of glycemia and lipid profiles.

We identified from patient engagement workshops that given the plethora of health-based apps currently available, a recommendation for the use of specific digital tools, such as Gro Health, should ideally be provided by a health care professional, with clear instructions on its optimal usage. In addition, patients provided invaluable insight into features they would like to see in any digital weight management tool. This is extremely important, given the recommendation of Topol Review that patients need to be included as partners (and encourage cocreation) when it comes to health technologies [19].

It is important to highlight that 63 people (32%) did not respond to our invitation, and it is not possible to conclude whether they were not interested, did not receive the right information, lost the letter, or forgot to reply. As a learning point from this, a landing page (web page) for the digital tool Gro Health was created. This provides all the necessary information about the digital tool, and it registers the interest of potential users.

For those participants who engaged with the Gro Health app, there was a high engagement and retention rate, similar to other reported studies using the intervention [13]. To improve future user engagement with digital health care apps, it is important that we learn from the existing literature within the field. In a meta-analysis by Szinay et al [20], the factors associated with higher uptake of a health-related app were availability at low cost, awareness of the app, and recommendations by clinicians. Factors associated with higher user engagement included user guidance, personalization, statistical data on progress, and self-monitoring features [20]. In a recent meta-analysis by Spaulding et al [21], although increased health app engagement was associated with improved weight and BMI, the authors suggested that further research is required to further understand mobile health user engagement in both inpatient and outpatient setting [21].

In recent years, there has been a substantial acceleration in the uptake and engagement with health-related apps, generally, reflective of the increasing digitalization of the health care delivery. This recent health care digitalization revolution has been catalyzed somewhat by the COVID-19 pandemic that has necessitated fundamental changes in the delivery of health care, including widespread implementation of remote appointments between patients and their health care teams. Our current health care digitalization revolution within the NHS offers huge potential for improvements in patient care and the efficiency of delivery of health care innovations. NHS obesity management is no exception. However, understanding the factors that predict disengagement with digital tools is important to optimize their future use and clinical utility within NHS-based clinical settings. Education of health care staff about the availability and benefits of digital health care tools is required to improve their uptake among patients, with clear instructions on their use and recommendations from a health care professional. A recent systematic literature review identified several sociotechnical factors that influence patients’ adoption of mobile health tools [22]. Some of the key findings from this comprehensive review were also seen as themes emerging from our patient engagement workshops, such as cost of the digital tools, incorporation into clinical pathways, and provision of appropriate health education and self-management.

This review provided a clear recommendation on a patient-centered approach that promotes patient adoption, with some of the key features such as fitting into patient’s overall treatment journey, inclusive design (especially for those users with less digital experience), comprehensive patient education and support, encouragement of the entire clinical team to use these tools, strong data ethics, and appropriate incorporation into health care policy [22].

Finally, we demonstrate the feasibility of the implementation of digital tool, Gro Health, to patients awaiting their first clinical appointment within our hospital-based obesity management team. Digital tools in weight management should not replace proper assessment and input from relevant health care professionals but rather augment traditional clinical care to optimize clinical efficiency in a novel, hybrid (blended) model of health care.

To fully embrace the digital health care revolution, its benefits, and huge potential, it is important that patients, their health care teams, and providers are involved in the creation of novel and bespoke digital health care tools for the future. As demonstrated from patient feedback, health care professional endorsement and patient cocreation are factors impacting any digital tool uptake. This highlights the importance of training and guidance for health care professionals to support patients with digital tools.

In addition, with so many digital tools available, it is key that tools demonstrate patient, clinician, and system benefits before adoption within health care systems. To this point, at the time of writing, Gro Health is the highest-rated digital health app as assessed by Orcha—reviewers of digital health apps on behalf of the NHS [23].

Digital tools such as Gro Health provide a foundation to support any unmet needs with education, behavioral support, and optimal user engagement that ultimately improves both the efficiency of health care delivery and, of course, patient outcomes. Future studies will assess the impact of such an initiative on patient-based outcomes and how these compare to traditional models in which there is usually very little or no patient contact and support from hospital-based clinical teams prior to their first clinical appointment.

### Limitations

This was an observational study with a relatively small number of participants. A formal power calculation was not performed. Additionally, it was not possible to completely assess the reasons for lack of interest among the third of eligible patients who did not respond to any contact.

### Conclusions

Emotional eating and higher BMI were associated with interest in the digital tool, Gro Health. Male gender was associated with reduced engagement with the app. There was no association between age or ethnicity and interest in the use of Gro Health app. Recommendations for the use of specific digital tools should ideally be provided by a health care professional, with clear instructions on its optimal usage. Additionally, patients should be involved in the cocreation of digital health tools.

Further research should evaluate the clinical impact of digital tools, such as Gro Health, as well as explore barriers and facilitators to engagement with digital tools in specialist weight management service.

## Appendix

Multimedia Appendix 1 Screenshots of the Gro Health app and key features.

## Abbreviations

COM-B: capability, opportunity, motivation, behavior
GAD-7: generalized anxiety disorder
NHS: National Health Service
PHQ-9: Patient Health Questionnaire-9
UHCW: University Hospitals Coventry and Warwickshire
