# Supplementary material for: Implementation of a Digital Health Tool for Patients Awaiting Input From a Specialist Weight Management Team: Observational Study
Source: JMIR Hum Factors. 2023 May 31;10:e41256. doi: 10.2196/41256 (PMC10267795; doi:10.2196/41256)

PATIENT APP

Gro Health

Available on the web, mobile (iOS, Android), smart speakers (Amazon, Google) and smart TV.

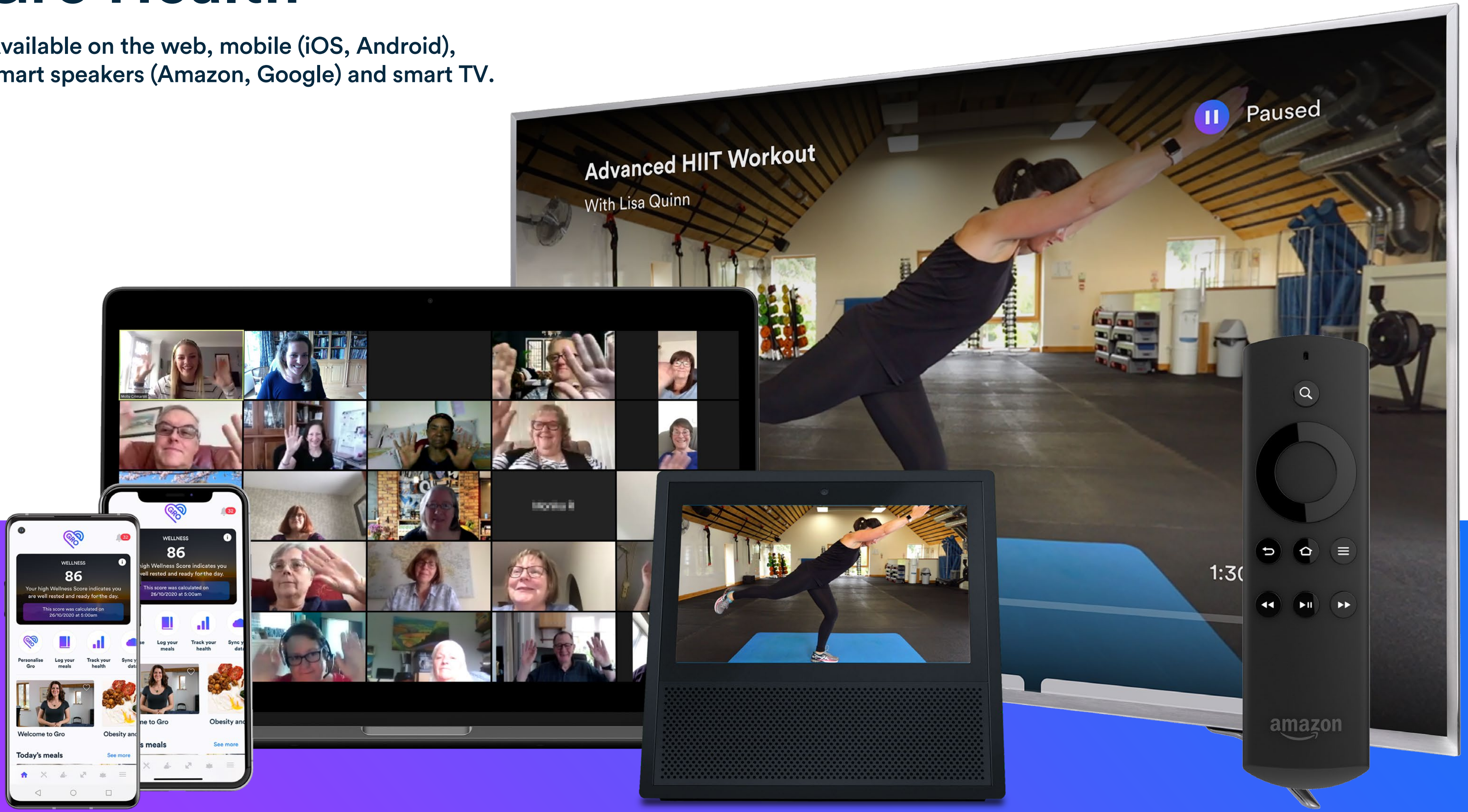

STRUCTURED EDUCATION

Education modules follow the same format

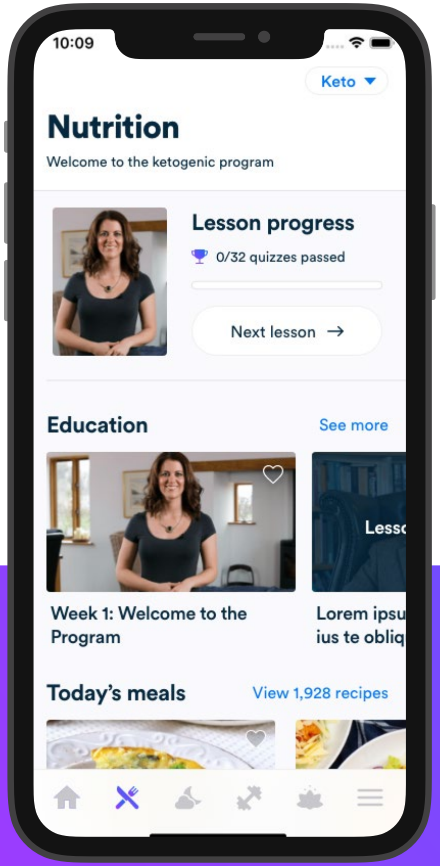

Home

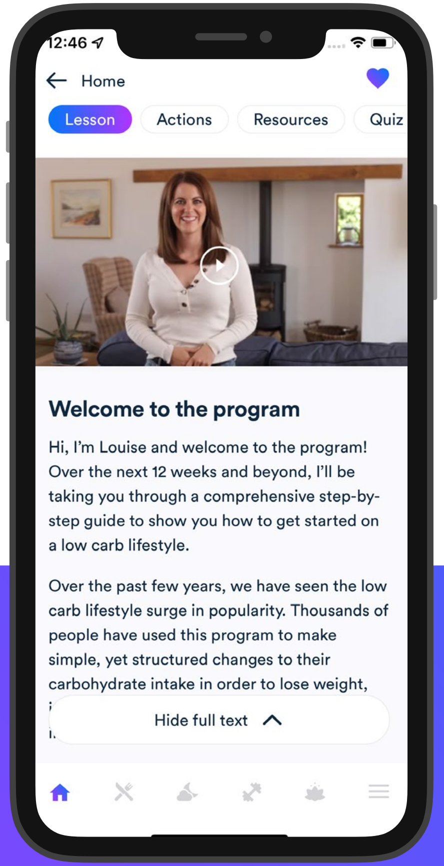

Education lesson

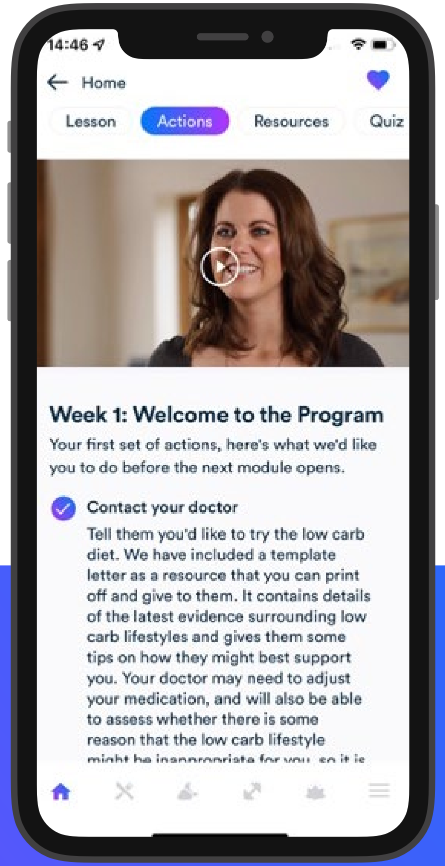

Action points

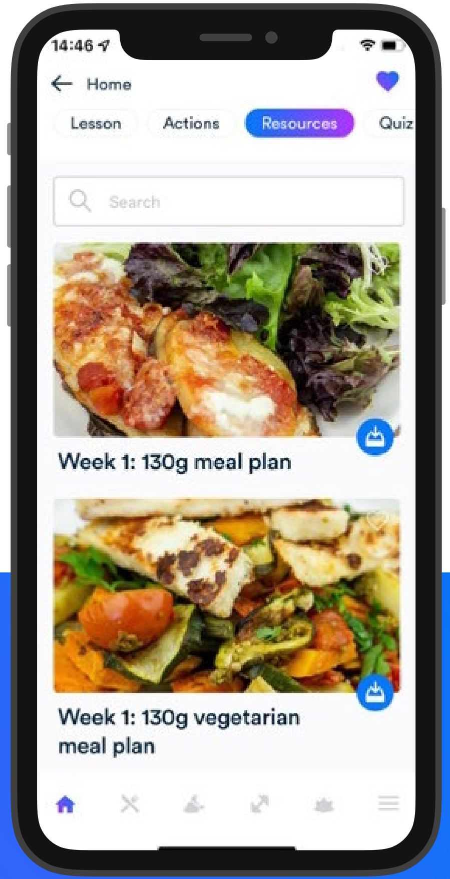

Resources

STRUCTURED EDUCATION

Education area

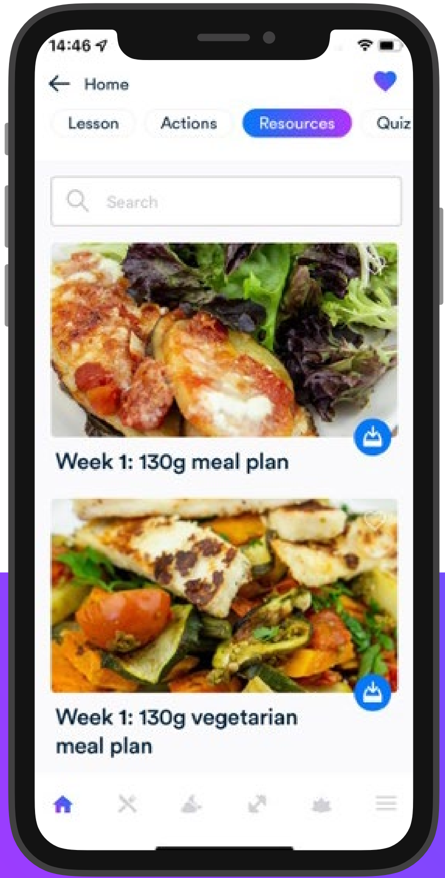

Resources

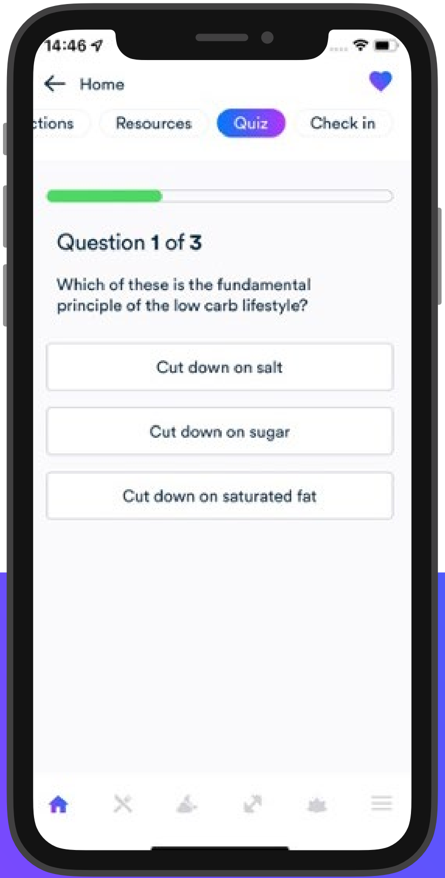

Quiz

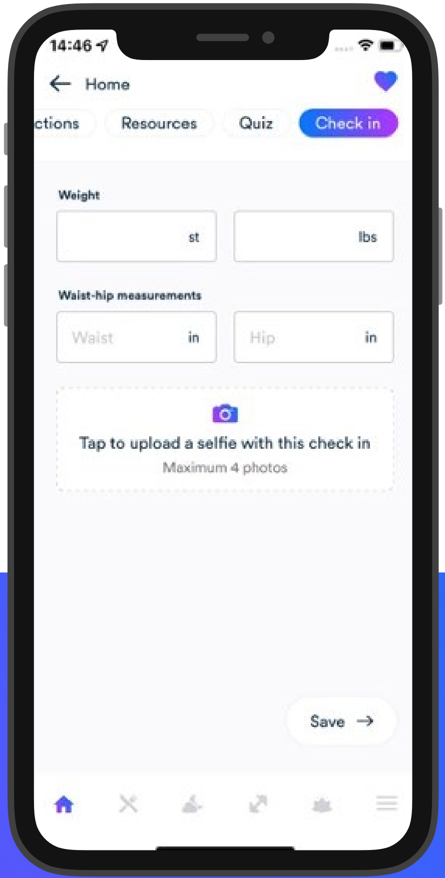

Check-in

PRECISION BEHAVIOURAL SUPPORT

Targeted, on-demand behaviour change features

Nutrition

Recipes, Live cook-a-longs, Meal plans, Shopping lists

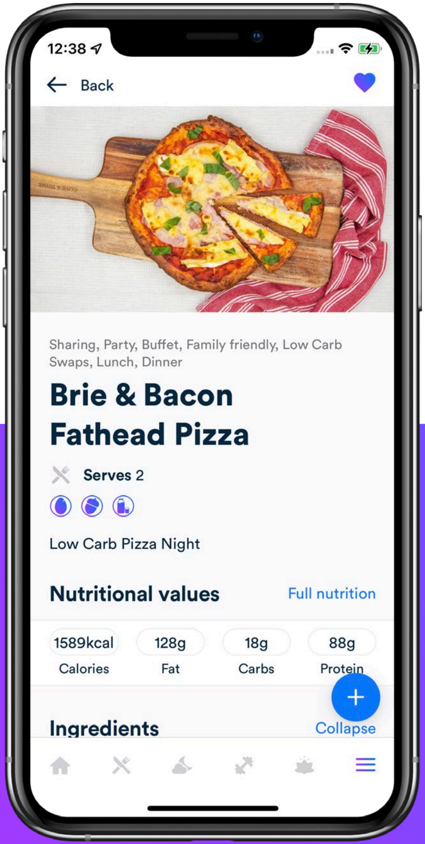

Mental Wellbeing

Immersive 360° mindfulness, Yoga, Guided meditations

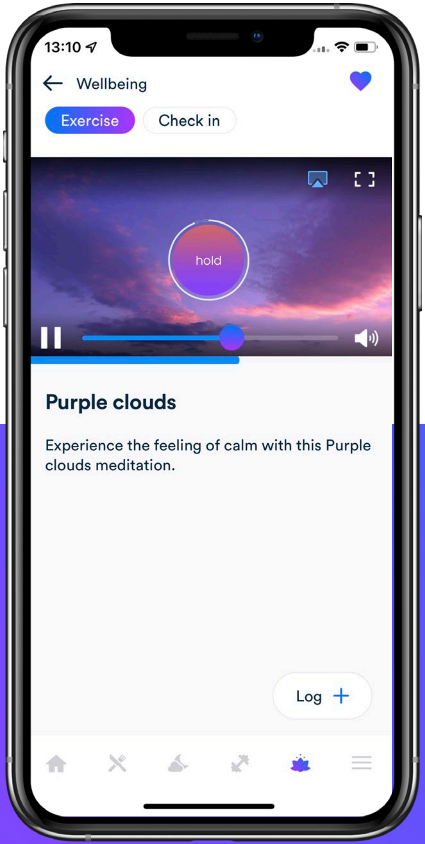

Activity

Tailored to fitness level: Cardio, HIIT, Tai Chi, Qi Gong and Stretching

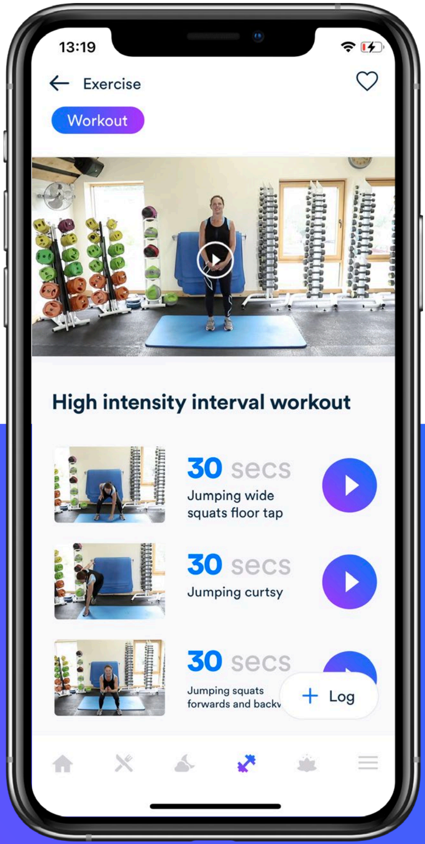

Sleep

Sleep stories, Meditations, Relaxation sounds

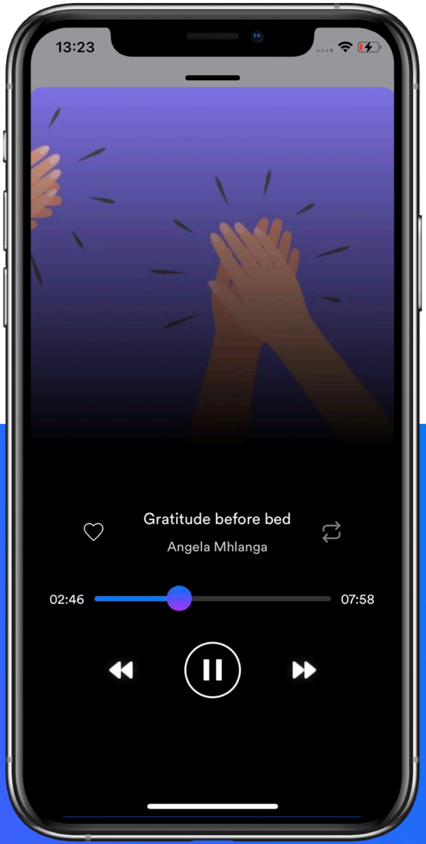

CLINICIAN APP

Real-time clinical dashboard and analytics

Real-time analytics for cohorts and individuals

Tailored user journey and notifications

Remote home monitoring

Bi-directional communication

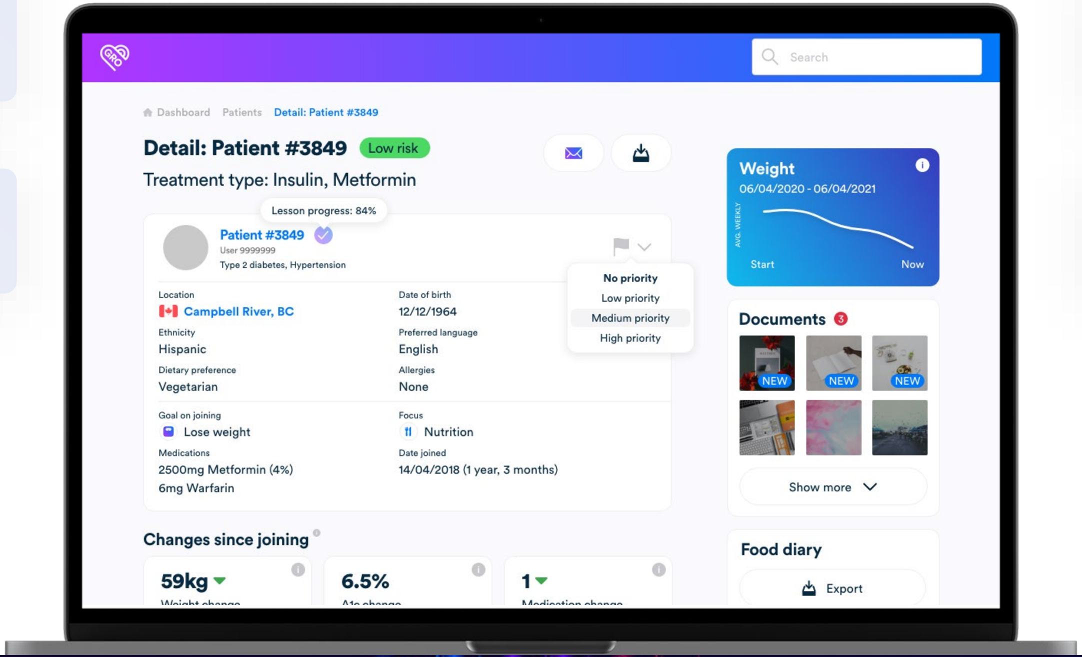

Supplement: Multimedia Appendix 1 [file humanfactors_v10i1e41256_app1.pdf]
